# Comparison of PTSD prevalence between immigrants and locals with psychotic disorders

**DOI:** 10.1192/j.eurpsy.2024.241

**Published:** 2024-08-27

**Authors:** A. Trabsa Biskri, A. Mané, R. Rodríguez, N. Zabaleta, L. Martínez, F. Casanovas, I. Ezquiaga, T. Legido, V. Pérez, B. Amann, A. Moreno

**Affiliations:** ^1^Psychiatry, Institut de Salut Mental, Hospital del Mar; ^2^Programa doctorat psiquiatría i medicina legal., Universitat Autònoma de Barcelona, Barcelona, Spain

## Abstract

**Introduction:**

Due to the global humanitarian crisis, there has been a significant increase in global immigration.(1)

The migration process typically involves multiple trauma exposures that are sustained over time(2), which may result in an impact on the mental health of these individuals(3), such as posttraumatic stress disorder(3). A recent meta-analysis estimated that 25% of migrants had PTSD(15), which is significantly higher than the 0.2% to 3.8 percent prevalence data found for the general population(4). In addition, a number of meta-analyses indicate an increased risk of psychosis among immigrants(5). Despite this rise, there is a gap in trauma research in non-refugee immigrants, particularly those with psychotic disorders.

**Objectives:**

To describe and compare PTSD diagnosis between immigrants and locals recruited from mental health services in Barcelona.

**Methods:**

Patients who have presented, according to DSM-V criteria, one or more non-affective psychotic episodes, were recruited in Acute and Chronic inpatients units at Hospital del Mar (Barcelona) from November 2019 to June 2021, leading to a total sample of 199 patients.

Demographic characteristics of patients, clinical data and main pharmacological treatment were recorded through a questionnaire. Database information was completed with electronic medical records. Global Assessment of Posttraumatic Stress Questionnaire (EGEP-5) was used as an instrument to assess PTSD diagnosis, main trauma nature and PTSD symptoms. Comparative analysis was performed with IBM SPSS Statistics (Chicago INC) using Chi-Square Test for qualitative variables and t-Student test for continuous variables. Covariate adjustment with demographic and clinical variables was performed by ANOVA test. Study received local ethics committee approval “CEIC” (No. 2019/8398/I).

**Results:**

From the total sample of 199 individuals, 98 were immigrants and 98 locals. From the total sample 39 individuals (19.69%) presented PTSD. 32.3% of the immigrants with psychotic disorders presented PTSD compared to 7.1% of the locals with psychotic disorders (F1=19.9, p=0.00). Most traumatic events related to PTSD in immigrants were: “murder of relatives” (33.1%), Physical violence (21.9%) and Terrorism (15.6%) in locals were: “physical violence” (28.6%). Immigrants and locals with psychotic disorders showed similar averages of symptoms, except for avoidance symptoms where locals showed a mean of 5.1 compared to a mean of 3.5 in the immigrant group. Finally, immigrants showed one more functionality affected area by PTSD (5.1) when compared to locals (4) (F7=3.9, p=0.05).

**Conclusions:**

According to our results there are important differences in PTSD prevalence between immigrants and locals with psychotic disorders. These findings ought to be taken into consideration for programs that are both clinically and sociopolitically tailored to improve assessment and treatment for this population.

**Disclosure of Interest:**

None Declared

